# Therapeutic resistance in cancer: microRNA regulation of EGFR signaling networks

**DOI:** 10.7497/j.issn.2095-3941.2013.04.003

**Published:** 2013-12

**Authors:** German G. Gomez, Jill Wykosky, Ciro Zanca, Frank B. Furnari, Webster K. Cavenee

**Affiliations:** Ludwig Institute for Cancer Research, University of California San Diego, La Jolla, CA 92093, USA

**Keywords:** Epidermal growth factor receptor, microRNA, tyrosine kinase inhibitors, therapeutic resistance

## Abstract

Receptor tyrosine kinases (RTKs) such as the epidermal growth factor receptor (EGFR) regulate cellular homeostatic processes. EGFR activates downstream signaling cascades that promote tumor cell survival, proliferation and migration. Dysregulation of EGFR signaling as a consequence of overexpression, amplification and mutation of the EGFR gene occurs frequently in several types of cancers and many become dependent on EGFR signaling to maintain their malignant phenotypes. Consequently, concerted efforts have been mounted to develop therapeutic agents and strategies to effectively inhibit EGFR. However, limited therapeutic benefits to cancer patients have been derived from EGFR-targeted therapies. A well-documented obstacle to improved patient survival is the presence of EGFR-inhibitor resistant tumor cell variants within heterogeneous tumor cell masses. Here, we summarize the mechanisms by which tumors resist EGFR-targeted therapies and highlight the emerging role of microRNAs (miRs) as downstream effector molecules utilized by EGFR to promote tumor initiation, progression and that play a role in resistance to EGFR inhibitors. We also examine evidence supporting the utility of miRs as predictors of response to targeted therapies and novel therapeutic agents to circumvent EGFR-inhibitor resistance mechanisms.

## Introduction

The human ErbB/epidermal growth factor receptor (EGFR) family is comprised of four members (EGFR/ErbB1, ErbB2, ErbB3 and ErbB4) that transduce signals upon binding to ligands to regulate important cellular processes such as cell division, differentiation, migration and programmed cell death^1^. Ligand binding induces EGFR dimerization, transphosphorylation of dimerized receptors and ultimately tyrosine kinase activation. Activated EGF-receptors recruit multiple adaptor and effector proteins and then initiate signaling through the PI3K, MAPK and STAT3 pathways to regulate a multitude of cellular activities^1^^,^^2^.

Since EGFR regulates fundamental cellular processes, it is not surprising that misregulation of EGFR signaling occurs frequently in several types of tumors including glioblastoma (GBM), colorectal cancer (CRC), head and neck squamous cell carcinoma (HNSCC), non-small cell lung cancer (NSCLC), breast, renal, ovarian, bladder, prostate and pancreatic cancers^3^^-^^5^. Consequently, multiple therapeutic agents and strategies have been developed to block the strong tumor promoting effects exerted by EGFR^6^. While some patients have shown encouraging responses to anti-EGFR therapies, durable responses are uncommon^2^. A thorough understanding of the factors that dictate response to EGFR inhibitors might spark the design of novel therapeutics to combat the development of resistance to such inhibitors.

MicroRNAs (miRs) are a novel group of non-coding small regulatory RNAs, which finely tune gene expression^7^ and are emerging as unique effector molecules of the different signaling cascades initiated by EGFR in normal and transformed cells. Here, we emphasize the role of the miRs most commonly involved in facilitating or suppressing aberrant EGFR signaling in a variety of tumor types. Additionally, we highlight the possibility of using miRs or anti-miR oligonucleotides as novel therapeutic agents to overcome resistance to anti-EGFR therapies. We also present evidence supporting the use of miRs and their targets as molecular predictors of response to EGFR inhibitors.

## Mechanisms of aberrant EGFR activation in cancer

Aberrant EGFR signaling occurs through a variety of mechanisms, including overexpression as a consequence of gene amplification, genetic mutations, cross-talk between mutant and wild-type EGFR, excessive levels of activating ligands or autocrine signaling and altered EGFR cellular localization.

### EGFR overexpression and gene amplification

Under normal physiological conditions, EGFR is present at about 4×10^4^ to 1×10^5^ EGFR molecules/cell^8^^,^^9^. In contrast, tumors can express about 5×10^5^ to 2×10^6^ EGFR molecules/cell^10^^-^^12^. In GBM, the EGFR gene is amplified to very high levels (>20 copies/cell)^13^ and in about 50% of primary GBMs where it is associated with poor prognosis^14^^-^^18^, as compared to secondary GBM patients^19^.

### Mutations

**Figure 1:**
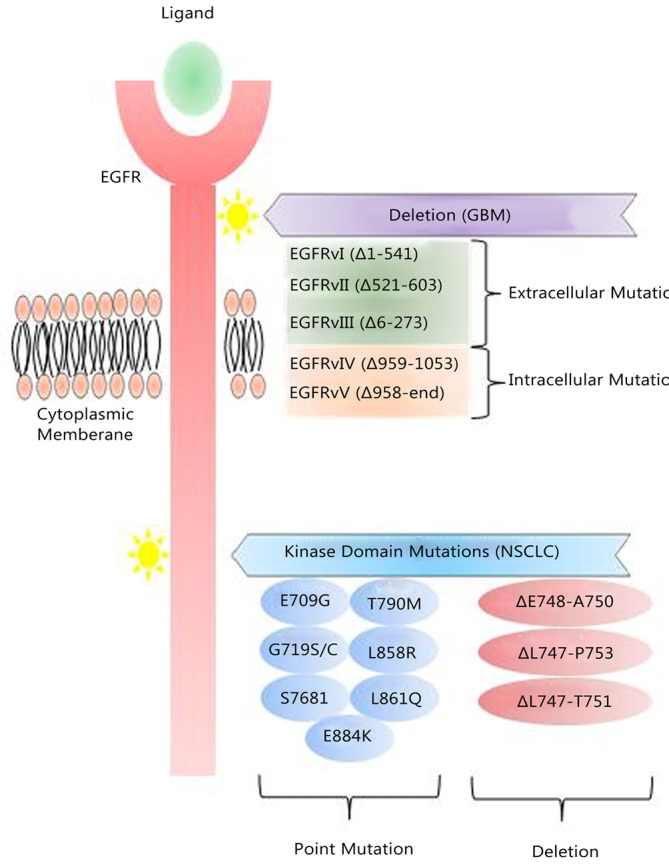
EGFR mutations in GBM and NSCLC. Tumor-type specific mutations of EGFR are well-established. In GBM, EGFR undergoes deletion processes that lead to the expression of truncated mutants, of which EGFRvIII is the major form that is associated with a poor response to conventional and EGFR-TKIs therapies. Point mutations of the EGFR kinase domain are predominant in NSCLC.

Various EGFR mutations are well-documented and shown to be tumor-type specific. One mutation with profound pathologic effects is a truncated form of EGFR, commonly found in about 50% of GBMs with EGFR amplification^9^, named EGFRvIII (also known as ΔEGFR, EGFR* or de2-7EGFR)^20^. EGFRvIII lacks a portion of the extracellular ligand-binding domain, is constitutively active and slowly recycled. EGFRvIII does not bind ligand but initiates constitutive mitogenic and cell survival signals^21^, and a resulting worse prognosis for GBM patient whose tumors express it^22^. Other truncation mutants of EGFR (EGFRvII; EGFRvV) have less demonstrated clinical relevance^23^ (**Figure 1**).

Point mutations in the intracellular portion of the EGFR gene have been found in different tumor types, in particular in the EGFR kinase domain in NSCLC (**Figure 1**)^24^^,^^25^. The L858R mutation and deletions in exon 19 confer constitutive kinase activity and are associated with a better response of lung tumors to EGFR kinase inhibitors^26^^-^^28^.

### Tumor microenvironment

Despite the strong tumorigenic effects exerted by EGFRvIII, EGFRvIII-expressing cells are not the predominant subpopulation in a brain tumor cell mass, which is often represented by wild-type EGFR expressing cells^29^. This phenomenon raised the possibility that EGFRvIII drives tumorigenesis in cooperation with wild-type EGFR. In a model of GBM heterogeneity, EGFRvIII-expressing cells produce secreted factors, such as IL-6 and LIF, which leads to cytokine co-receptor gp130-wild-type EGFR cross-talk and subsequent transactivation of wild-type EGFR to promote tumorigenesis^30^. Interestingly, hypoxia has been proposed to enhance EGFR-mRNA translation, thus providing another mechanism of increased EGFR expression and signaling^31^.

### Ligand-mediated activation

EGFR contains four extracellular domains (DI, DII, DIII and DIV), among which DI and DIII are required for ligand binding^32^. Ligand binding causes EGFR to form homodimers, or heterodimers with other ErbB family members (ErbB2, ErbB3, ErbB4), thus leading to tyrosine kinase domain activation and signal transduction^5^. Under physiological conditions, many ligands bind and activate EGFR, including epidermal growth factor, transforming growth factor-alpha, heparin-binding EGF, betacellulin, epigen, epiregulin, amphiregulin and neuregulin 2b^33^. Different ligands might have different downstream effects, but the mechanism remains unclear^34^. Ligand overexpression in cancer leads to persistent EGFR signaling^35^^,^^36^.

### Cellular localization

Membrane-associated EGFR signaling is augmented by heterodimerization with other members of the EGFR family, such as ErbB2^37^. Importantly, EGFR signaling is not completely restricted to the plasma membrane. EGFR localizes to the nucleus of cancer cells and its nuclear localization is associated with a poor prognosis in different types of cancers^38^^-^^45^. Nuclear EGFR appears to promote cell proliferation through its tyrosine kinase activity or by acting as a transcriptional regulator^46^^,^^47^. EGFR has also been found in the mitochondria^48^, thus providing a new perspective on EGFR subcellular location and its role in cancer.

## EGFR-targeted therapies

As EGFR promotes oncogenesis by activating the signaling pathways that regulate tumor formation and progression, it is not surprising that it has become one of the most heavily targeted molecules for therapy. The three most common agents for targeting EGFR are monoclonal antibodies (mAbs), vaccines, and small molecule inhibitors.

### Antibodies

The mAbs cetuximab^49^^-^^51^ and fully humanized panitumumab^52^^,^^53^ are specific for the extracellular domain of EGFR and display anti-tumor activity in patients. These antibodies, which are FDA-approved for use in the U.S., bind to EGFR and prevent ligand-mediated activation of the receptor. Another antibody, mAb 806, binds not only to amplified wild-type EGFR but also more strongly to the mutant EGFRvIII^54^^,^^55^. This antibody is advantageous because the mutant receptor is not expressed in normal tissue, so should minimize side effects resulting from antibody binding to EGFR expressed on non-tumor cells. While the primary anti-tumor activity of EGFR mAbs is attributed to blocking receptor activation, other mechanisms likely contribute to the anti-tumor effects such as receptor downregulation^56^ and antibody-dependent cellular cytotoxicity (ADCC)^57^.

### Anti-EGFR vaccines

Another approach for targeting EGFR is vaccines that elicit an immune response against EGFR-expressing tumor cells. One vehicle is dendritic cells pulsed with EGFR-specific antigenic peptides^58^. CDX-110 is a peptide vaccine that induces anti-tumor immune responses to EGFRvIII positive cells; this vaccine has shown pre-clinical efficacy and some encouraging clinical results^59^. An alternate approach which is becoming increasingly more common is the engineering of T lymphocytes to express chimeric antigen receptors (CARs). EGFR-targeted CAR T cells have demonstrated anti-tumor efficacy both *in vitro* and *in vivo* with low systemic toxicity^60^.

### Small molecules

Small molecule inhibitors that compete with ATP for binding to the active conformation of the EGFR kinase domain are perhaps the most widespread approach to targeting this receptor^61^. The orally bioavailable reversible inhibitor, erlotinib, is FDA-approved for the treatment of NSCLC, and its closely related cousin, gefitinib, is approved for multiple solid tumors in countries outside the U.S. Afatinib is a second-generation, irreversible EGFR/ErbB2 inhibitor that has recently gained FDA approval for EGFR-mutant NSCLC along with a companion diagnostic test to determine the EGFR mutation status^62^^,^^63^.

## Mechanisms of resistance to EGFR inhibitors

While these targeted therapeutic approaches are rational strategies, clinical benefit from them is rare. Response is typically observed in only a subset of patients, especially in an unselected patient population. Thus, the decision to begin and continue treatment must be based upon reliable biomarkers that are predictive of response. Specific mutations in the kinase domain of EGFR render some NSCLC tumors exquisitely dependent on EGFR-mediated signaling for survival and are predictive of response to EGFR-tyrosine kinase inhibitors (TKIs)^26^^-^^28^. In fact, the presence of these mutations in NSCLC tumor biopsy tissue is now required in order for a patient to receive TKI therapy. In nearly all other tumor types, however, there does not appear to be a specific EGFR mutation that predicts for response to these therapies, and it has become clear that expression of the target alone does not suffice as a predictive biomarker^64^. Rather, it appears that more complex mechanisms underlie the response of tumors to EGFR-targeted therapy.

Resistance to EGFR-targeted therapy following an initial clinical response to these drugs is a common clinical observation. Although mechanisms, such as the presence of mutations in codons 12 and 13 of the K-Ras gene^65^ exist that render tumor cells inherently resistant to EGFR-targeted therapies, acquired resistance is a more widespread clinical problem. Thus, the mechanisms of and methods to overcome resistance are an area of intense study. In the case of EGFR-targeted antibodies and vaccines, resistance almost always manifests as the outgrowth of a population of tumor cells that are devoid of EGFR expression^66^ meaning that they have “escaped” the targeted therapy by eradicating the target.

### T790M EGFR mutation

Treatment of NSCLC with the EGFR-TKIs gefitinib and erlotinib results in the generation of a second-site mutation in the EGFR kinase domain that changes a threonine to a methionine—T790M—in at least 50% of patients^67^. This mutation increases the affinity of the kinase domain for ATP while decreasing affinity for the small molecules and the consequent decrease of drug binding causes sustained phosphorylation of EGFR and clinical drug resistance. One approach to circumvent T790M-mediated resistance has been the use of irreversible small molecule inhibitors such as afatinib, which has demonstrated activity against T790M mutant cells and tumors in preclinical models^68^. Disappointingly, this has resulted in only a modest effect in patients who progressed during prior treatment with erlotinib or gefitinib^69^.

### Alternative receptor tyrosine kinase activation

While the T790M mutation represents a direct mechanism for drug resistance that occurs at the level of drug accessibility to the target, there are a number of indirect mechanisms that involve the increased expression or activation of alternative growth factor receptors to maintain signal flux, despite EGFR inhibition. Perhaps the best characterized of these is the amplification of the Met receptor^70^ and/or elevated levels of its ligand HGF^71^. In gefitinib resistant NSCLC cell lines, Met drives ErbB3-dependent activation of the PI3K pathway^72^. In addition, increased expression and activity of Met and ErbB3 are associated with resistance to cetuximab^73^. The receptor Axl was also identified as a potential target for overcoming EGFR inhibitor resistance associated with epithelial to mesenchymal transition^74^. Interestingly, HGF has recently been shown to drive resistance through stimulation of EGFR binding to Axl and the EphA2 receptor in a kinase-independent fashion^75^.

### PI3K activation and PTEN inactivation

PI3K signaling pathway activation is a frequently observed mechanism of resistance. One way to achieve elevated levels of PI3K signaling and resistance to targeted EGFR inhibition is by direct mutation of *PIK3CA*, the gene encoding the p110 catalytic and p85 regulatory subunits of PI3K. In colorectal tumors, *PIK3CA* mutations are associated with reduced sensitivity to cetuximab^76^. In GBM, patients who responded to EGFR-TKIs demonstrated co-expression of the mutant EGFRvIII and PTEN, a negative regulator of PI3K activity^77^. It appears that for EGFR targeting to be effective, complete inhibition of PI3K activity must be achieved. Tumors lacking PTEN expression generally have elevated and sustained PI3K pathway activity, and restoration of functional PTEN re-sensitizes resistant cells to erlotinib^78^. Furthermore, EGFR-TKI resistance is associated in some cases with FGFR and Src family kinase-mediated phosphorylation of PTEN at tyrosine 240^79^. Similar persistent signaling can be achieved by the co-activation of other RTKs such as PDGFR^80^.

Some of the scenarios mentioned above point toward rational strategies for targeting that take advantage of new therapeutic vulnerabilities arising as a result of the specific resistance mechanism. Combination therapies or salvage therapies utilizing other RTK inhibitors or specific inhibitors to pathway components that contribute to resistance are being explored, both pre-clinically and clinically. One striking example is the induction of the promyelocytic leukemia (PML) gene following EGFR-TKI-mediated inhibition of mTOR signaling^81^. Expression of PML confers sensitivity to arsenic trioxide and points toward a novel therapeutic strategy for resistant tumors with elevated PML.

## MicroRNAs regulate EGFR signaling and susceptibility to EGFR inhibitors

### MicroRNAs

MicroRNAs (miRs) are a group of non-protein encoding RNAs that are 19-25 nt in length and block translation or facilitate mRNA degradation upon binding to complementary sequences in the 3' UTR of their target mRNAs^7^. The first miR, lin-4, was discovered 20 years ago where it was shown to decrease lin-14 protein expression in *C. elegans*^82^. Subsequent studies identified the existence of new miRs in several species including mice and humans^83^^-^^85^ and approximately 900 miRs have been so far identified^86^.

MiRs are transcribed by RNA polymerase II as large primary transcripts (pri-miRs) that are processed by Drosha/DGCR8 complexes to yield 60-110 nt long hairpin containing-precursor miRs (pre-miRs)^87^. After export of the pre-miRs to the cytoplasm by exportin-5^88^, mature miRs are excised from the pre-miRs by the Rnase III enzyme, Dicer^89^, and loaded into the RNA-induced silencing complex (RISC)^90^. There, mature miRs are guided to their appropriate target mRNAs to prevent translation. A recently discovered alternate and conserved miR biogenesis pathway, the miRtron pathway, generates pre-miRs through splicing mechanisms that do not require Drosha/DGCR8 activity^91^^,^^92^.

Ample evidence indicates that the de-regulation of miR expression and activity, as a consequence of genomic alterations^93^, miR gene methylation^94^, aberrant transcription^95^ and defective miR processing^96^^,^^97^, are intimately involved in cancer initiation, maintenance, and progression^7^. Below we focus on the regulation of EGFR signal networks by miRs in cancer and the involvement of miRs in facilitating resistance to EGFR-inhibition (**Table 1**).

**Table 1:** MicroRNAs that sustain or repress EGFR signaling

MiR	MiR regulator	Targets
MiR-21	EGFR^98^, ErbB2^99^, c-MET^100^, AP-1^101^	PTEN^100^^,^^102^, SPRTY^103^^,^^104^, PDCD4^101^^,^^104^
Let-7	EGFR^105^, C-Myc^106^, LIN28^107^^,^^108^	Ras^109^, C-Myc^108^
MiR-7	EGFR^110^^,^^111^	EGFR^112^^,^^113^, IRS1^112^^,^^114^, IRS2^112^, RAF-1^114^^,^^115^
MiR-34	p53	c-MET
MiR-221/222	EGFR^100^, c-MET^100^	PTEN^100^, Apaf-1^100^
MiR-30b/c	EGFR^100^, c-MET^100^	BIM^100^

### MiR-21

MiR-21 is a bona-fide “oncomir” and one of the most widely studied miRs due to its dramatic upregulation in many cancers, ability to target the tumor suppressor PTEN and thereby reducing tumor susceptibility to TKIs^77^, as well as its regulated expression in hypertrophic heart disease models^116^. Significant overexpression of miR-21 was observed in primary GBM specimens and cultured cells relative to normal brain tissues and cells. Moreover, functional inhibition of miR-21activity using 2′-*O*-methyl and locked nucleic acid oligonucleotides revealed miR-21 to be an anti-apoptotic miR^117^. Subsequent studies have focused on clarifying the mechanisms of miR-21 deregulation in cancer, identifying miR-21 targets and their involvement in therapeutic response and the potential of miR-21 to serve as a novel cancer target.

**Figure 2:**
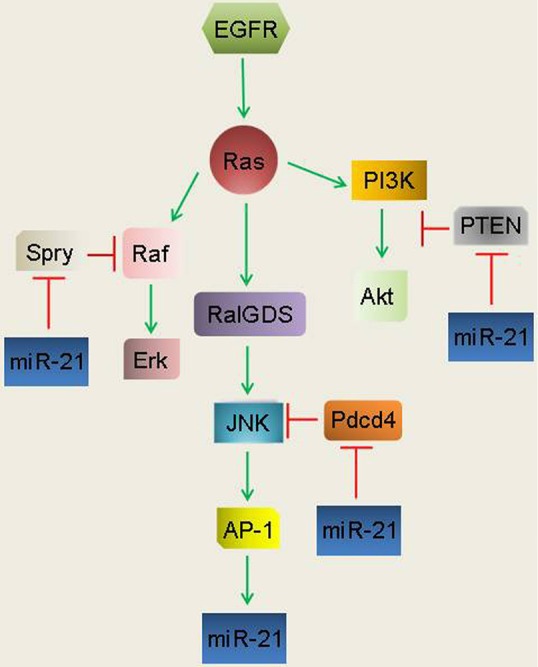
EGFR induces miR-21 to inhibit negative regulators of downstream EGFR pathways. Induction of miR-21 occurs upon AP-1 activation in response to EGFR/RalGDS/ JNK signaling. MiR-21 targets the PDCD4 and PTEN tumor suppressors to achieve maximal Ras/RalGDS/JNK/AP-1 and Ras/PI3K/Akt signaling. MiR-21 targets the ligand-induced negative RTK feed-back regulator, SPRY, to maintain prolonged Ras/Raf/Erk signaling.

In GBM, inhibition of miR-21 activity increased PTEN expression and decreased tumorigenicity, EGFR expression and Akt activation^102^. MiR-21 is positively regulated by EGFR in cancer cells as demonstrated by the finding that AG1478, an EGFR-TKI, blocked EGFR induction of miR-21^98^. Interestingly, activation of the EGFR family member, ErbB2, induced miR-21 to promote cell invasion through suppression of the well-established miR-21 target, PDCD4^99^. Critically, oncogenic HRas^G12V^ was sufficient to induce miR-21^99^, consistent with oncogenic Ras mutants conferring resistance to EGFR-TKIs^65^. The discovery of a novel autoregulatory loop revealed that miR-21 targets PDCD4, a negative AP-1 regulator, upon its induction by AP-1 in response to Ras signaling^101^, and several other studies confirmed that miR-21 is positively regulated by Ras/ERK signaling^103^^,^^118^^,^^119^. Interestingly, data from transgenic tumor models show that miR-21 drives tumorigenesis by repressing negative regulators of the Ras/MEK/ERK, Ras/PI3K/Akt and Ras/RalGDS/JNK pathways, thus further demonstrating that miR-21 acts as an effector of Ras to promote transformation^103^^,^^104^. Collectively, these reports suggest that EGFR family members positively regulate miR-21 as a means to achieve a signaling threshold required for transformation and the maintenance of malignant cellular phenotypes (**Figure 2**).

**Figure 3:**
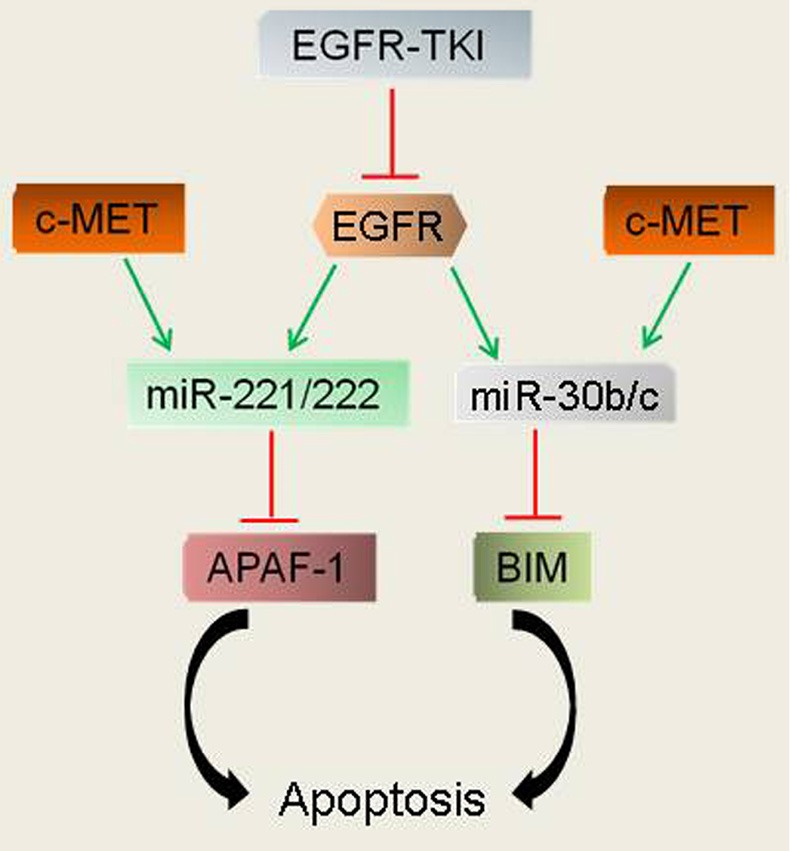
A model of resistance to EGFR-TKIs. EGFR induction of the miR-221/222 and miR-30b/c family members represses the pro-apoptotic genes Apaf-1 and BIM, rendering cells less susceptible to apoptotic cell death. EGFR-TKIs reverse the suppression of Apaf-1 and BIM to induce apoptosis of EGFR-dependent tumor cells. Amplication and/or activation of c-MET restores the induction of miR-221/222 and miR-30b/c and, consequently, the repression of Apaf-1 and BIM to escape EGFR-TKI-mediated apoptosis.

The ability of miR-21 to inhibit apoptosis and sustain the activation of oncogenic signaling pathways led to the proposal and subsequent demonstration that its expression could predict and modify responses to conventional cancer therapies^120^^,^^121^. With regard to resistance to EGFR-TKIs, miR-21 blockade is able to reverse the EMT phenotype associated with EGFR-TKI resistance^74^^,^^122^^,^^123^. In human breast cancer models miR-21 upregulation caused acquired resistance to the anti-HER2/neu antibody, Trastuzumab^124^. Trastuzumab-resistant cells showed decreased PTEN expression that was restored upon inactivation of miR-21. When combined with Trastuzumab, miR-21 anti-sense oligonucleotide therapy significantly inhibited the growth of Trastuzumab-resistant tumors. Importantly, miR-21 overexpression was correlated with reduced PTEN levels in Trastuzumab-resistant breast cancer patients^124^. Gefitinib-resistant lung cancer cells having high levels of miR-21, miR-30b/c and miR-221/22, as consequence of c-MET overexpression, were rendered susceptible to gefitinib when treated with SU11274, a c-MET inhibitor^100^. Combined EGFR and c-MET inhibition induced PTEN, Apaf-1 and BIM, as a consequence of downregulation of miR-21, miR-30b/c and miR-221/221^100^. Collectively, these reports suggest that EGFR and c-Met coordinately regulate multiple miRs to fine-tune downstream signaling cascades that render tumors resistant to EGFR-TKIs (**Figure 3**).

### Let-7

The let-7 family members are highly conserved and reside in regions of the human genome frequently altered in cancers^93^^,^^125^. Let-7 was first shown to behave as a tumor suppressor in a study where it inhibited lung cancer growth^126^. In addition, the observed downregulation of let-7 family members correlated with shorter survival in a cohort of 143 lung cancer cases. The mechanism of tumor suppression by let-7 was revealed when HRas, NRas and KRas were shown to be let-7 targets^109^ and that let-7 suppression was necessary for tumor initiation, maintenance and metastasis^114^^,^^127^.

The frequent suppression of let-7 in cancers prompted studies to identify the mechanisms regulating its stability and biogenesis. The demonstration that the LIN28 RNA binding protein selectively impairs processing of the let-7 family led to the verification that inhibition of let-7 processing by LIN28 and LIN28B is sufficient to transform cells^107^^,^^108^. Furthermore, LIN28 is overexpressed in a subset of human cancers where it induces expression of two let-7 targets, KRas and c-Myc, to transform NIH3T3 cells. Interestingly, c-Myc positively regulates LIN28B to repress let-7 family members^106^, suggesting the possibility that an RTK/Ras/ERK/C-Myc signaling loop is ultimately responsible for let-7 inactivation. Supporting this hypothesis, SHP2, a protein tyrosine phosphatase required for maximal ERK activation downstream of grow factor receptors, activated c-Myc, and this resulted in the repression of let-7 and promotion of breast cancer maintenance^128^. These interactions were underscored by finding that inhibition of EGFR kinase activity with gefitinib induced let-7c, showing that EGFR signaling suppresses let-7^105^.

Mutant forms of the Ras proteins occur in certain tumors where they confer protection to cytotoxic therapies^65^. Experimental manipulation of the LIN28-let-7-KRas regulatory network by let-7 overexpression and LIN28 silencing caused radio-sensitization of KRas mutant lung and pancreatic cancer cells^129^, suggesting that let-7 and KRas might serve as markers of survival and therapeutic response. In patients with KRas-mutated mCRC, high let-7a expression was predictive of better overall and progression-free survival^130^. Interestingly, while low let-7 expression and KRas mutation expectedly predict poor survival^126^^,^^131^, a variant KRas, KRas-LCS6, is associated with increased risk of developing NSCLC and reduced survival in HNSCC^132^^,^^133^. The KRas-LCS6 allele bears a single nucleotide polymorphism (SNP), is present in about 20% of NSCLC tumors analyzed and disrupts let-7 binding to the 3’UTR of KRas. DNA sequencing of KRas in mCRC tissues obtained from patients that had undergone salvage irinotecan-cetuximab therapy showed that KRas-LCS6 positive tumors responded poorly to the therapy relative to tumors without the LCS6 SNP^134^. These initial observations were supported by other results that linked the KRas-LCS6 with non-response to anti-EGFR therapy in tumors bearing wild type KRas and BRAF^135^. Collectively, these reports suggest that the disruption of the let-7-KRas regulatory network by the KRas-LCS6 SNP is predictive of response to anti-EGFR therapy in CRCs with wild type KRas and BRAF.

### MiR-7

MiR-7 was first implicated as an effector of EGFR signaling in studies of *Drosophila* where inactivation of the Yan transcription factor through the EGFR/ERK signaling axis induces miR-7 to promote photoreceptor differentiation^110^. In NSCLC, miR-7 induction by both wild type and mutant EGFR L858R required the RAS/ERK/c-Myc signaling axis^111^ to promote lung tumorigenesis by repressing the transcriptional regulator ERF. Supporting the oncogenic role of miR-7, molecular diagnostic testing showed that 60% of NSCLC fine-needle aspirates had upregulation of miR-7^136^. MiR-7 was also significantly upregulated in renal cell carcinoma (RCC) samples relative to normal tissues and miR-7 was required to promote RCC survival, proliferation and migration^137^.

Paradoxically, several studies have shown that miR-7 also acts as tumor suppressor by directly targeting EGFR itself. However, the categorization of miRs as tumor suppressors or oncogenes must take into account the cellular context as this dictates the functions of miRs^138^. It was first demonstrated in GBM that miR-7 targets EGFR and that impairment of miR-7 processing leads to its downregulation^112^. In addition, miR-7 inhibited GBM cell proliferation, survival and migration while also inhibiting Akt signaling by targeting insulin receptor substrates, IRS-1 and IRS-2. MiR-7 also attenuates the activation of Akt and ERK that is induced by EGFR signaling in multiple cancer cell types^113^. In addition to downregulating EGFR, miR-7 targets several other genes involved in EGFR signaling and tumorigenesis, indicating that miR-7 negatively regulates EGFR signaling in several types of cancers. Subsequent studies confirmed that miR-7 inhibits EGFR, and its downstream signaling components, to negatively regulate tumor cell migration, invasion, metastasis and tumorigenesis in various tumor cell types^139^^-^^141^. Collectively, these observations provided the impetus to target EGFR signaling networks using miR-7 to circumvent resistance to conventional and targeted therapies.

*In vitro* radiosensitivity experiments were employed to determine the ability of miR-7 to reverse the radioresistance conferred on human cancer cells by EGFR^142^. As would be predicted from the prior studies cited above, miR-7 blunted EGFR/PI3K/Akt signaling and reversed the radio-resistance. Correspondingly, direct injection of a liposome-encapsulated miR-7 plasmid into established EGFR-TKI sensitive and resistant tumors bearing the T790M EGFR mutant resulted in significant tumor regression in conjunction with repression of EGFR, RAF-1 and IRS-1 expression^142^. In head and neck cancer, miR-7 functioned in a synergistic manner with erlotinib to render FaDu Erlotinib-resistant cells susceptible to the growth inhibitory activities of the drug^115^. Interestingly, expression profiling analysis suggested that the downregulation of RAF-1 and EGFR and its ligand TGF-α by miR-7 was a possible mechanism by which miR-7 orchestrates the inhibition of EGFR signaling at multiple levels115. In total, these observations show promise for miR-7-based strategies to effectively target EGFR addicted and EGFR-TKI resistant tumors.

### MiR-34

The p53 tumor suppressor senses DNA damage, cellular stress and inappropriate mitogenic cues. In response to such signals, p53 facilitates DNA repair, induces cell death and arrests cell division^143^. The p53 pathway requires the induction and activation of many gene products to regulate a diverse array of cellular stress responses. In 2007, it was shown that p53 induces expression of members of the miR-34 family in response to ionizing radiation^144^. The regulation of miR-34 family members by p53 and their involvement in p53-induced cell death and cell cycle arrest was then validated in other model systems^145^^-^^147^.

Of relevance to EGFR-TKI resistance, as c-Met overexpression confers resistance to EGFR-TKIs^70^, miR-34 directly targeted cell cycle-related proteins and c-Met in MEF cells^144^. In GBM and ovarian cancer, miR-34a/b/c expression was inversely correlated with c-Met expression^148^^,^^149^. In addition, miR-34 inhibited cell invasion, proliferation and tumorigenesis, while c-Met overexpression partially reversed the cell death and cell cycle arrest induced by miR-34^149^^,^^150^. C-Met was established as a bona fide miR-34 target in melanoma, lung, colon, breast and gastric cancer cells^151^. Importantly, miR-34 inhibited activation of c-Met, Akt, ERK and it impaired c-Met driven invasion.

Given c-Met’s ability to promote tumor cell motility, invasion and resistance to EGFR-TKIs, c-Met inhibition has been hypothesized to limit tumor spreading and resistance to cytotoxic agents and targeted therapies^152^. In support, the induction of miR-34 by p53 downregulated c-Met and inhibited c-Met mediated tumor cell motility and invasion^153^. Combined treatment of hepatocellular carcinoma cells with miR-34a and the c-Met inhibitor, SU11274, significantly induced cell death and inhibited cell proliferation^154^. To explore the feasibility of miR-34a as an anti-tumor agent, the ability of miR-34 lenti-virus to suppress the growth of therapeutically resistant lung cancers bearing KRas and p53 mutations was determined^155^. As anticipated, KRas/p53 mutant tumors showed decreased miR-34 expression and increased c-Met levels relative to normal lung and lenti-viral delivery of miR-34 impaired lung tumor initiation and progression. These findings suggest that miR-34 replacement therapies might sensitize resistant tumors to EGFR-TKIs by suppressing c-Met expression and its activation of oncogenic signaling pathways.

## Conclusion

There is a rapidly increasing understanding of EGFR signaling, therapeutic targeting and mechanisms of resistance. There is also the accumulation of a large body of knowledge about miRs and their intimate involvement in tumorigenesis and tumor progression. This has led to the recognition of mechanisms by which tumors modulate miR activities to thrive when subjected to selective pressures applied by therapy. It is evident that miRs represent a novel group of regulatory RNAs that EGFR impinges upon to promote its tumorigenic activities. The ability to modulate miR activity and/or the activity of miR targets suggests a novel therapeutic avenue to overcome resistance mechanisms to conventional and EGFR-targeted therapies.
